# Does Ossein–Hydroxyapatite Complex Improve the Results of Lower-Leg Lengthening Osteotomies with the Ilizarov Method?

**DOI:** 10.3390/life16081244

**Published:** 2026-07-27

**Authors:** Piotr Morasiewicz, Monika Zaborska, Łukasz Tomczyk, Katarzyna Sznajder, Igor Kowal, Krystian Kazubski

**Affiliations:** 1 Department of Orthopaedic and Trauma Surgery, Institute of Medical Sciences, University of Opole, Al. Witosa 26, 45-401 Opole, Poland; 2 Faculty of Medicine, Institute of Medical Sciences, University of Opole, Ul. Oleska 48, 45-052 Opole, Poland; 3 Department of Food Safety and Quality Management, Poznan University of Life Sciences, Wojska Polskiego 31, 60-624 Poznań, Poland; 4 Clinical Department of Imaging Diagnostics, Institute of Medical Sciences, University of Opole, Al. Witosa 26, 45-401 Opole, Poland; 5 Department of Orthopaedic and Trauma Surgery, Multidisciplinary Hospital in Zgorzelec, Lubańska 11-12, 59-900 Zgorzelec, Poland

**Keywords:** osteogenon, osteotomies, Ilizarov method, ossein, hydroxyapatite, ossein–hydroxyapatite complex

## Abstract

**Background:** Good Ilizarov treatment outcomes are associated with low complication rates, rapid formation of strong bone regenerate, and relatively early removal of the Ilizarov external fixator. Some authors recommend the use of pharmaceutical agents that facilitate and accelerate bone union. There are no previous studies evaluating Osteogenon in Ilizarov osteotomies. The purpose of our study was to assess radiological and clinical effects of Osteogenon in patients undergoing osteotomy combined with Ilizarov fixation. **Methods:** In this retrospective study we assessed 35 patients who had undergone lower leg lengthening osteotomies with the Ilizarov method in the years 2021–2022 and received adjunctive Osteogenon. The control group comprised 60 patients matched for age, sex, BMI, and comorbidities, who did not receive Osteogenon. We assessed the following clinical and radiological parameters: the duration of fixation, total initial limb shortening, total limb lengthening, achieving bone union following osteotomy, elongation index, alignment index, complications, analgesic use, and satisfaction with treatment. **Results:** The median duration of fixation was worse (longer) in the Osteogenon group (183 days) compared to the control group (143 days), *p* = 0.014. There were no differences between groups in initial limb shortening, median limb lengthening, median elongation index, median alignment index, and complication rate. A proportion of 22.9% of patients in the Osteogenon group and 80% patients in the control group took analgesics on post-treatment follow-up, *p* < 0.001. In the study, 65.71% of Osteogenon patients and 55% of control patients were very satisfied with the treatment. **Conclusions:** Taking Osteogenon improves patient’s satisfaction with treatment and limits analgesic use following osteotomies combined with Ilizarov fixation. Osteogenon was associated with a significantly longer external fixation period and failed to demonstrate measurable improvements in radiological outcomes. Any considerations regarding the potential benefits of using Osteogenon should be treated with caution.

## 1. Introduction

Osteotomies are a popular method of treating limb shortening and other limb deformities [1,2,3,4]. One of the most popular osteotomy techniques involves the Ilizarov fixation method [4,5,6,7,8,9,10]. The use of this technique allows for new bone tissue (i.e., bone regenerate) formation following controlled osteotomy performed as part of limb lengthening or deformity correction; this process is similar to callus formation following bone fracture [1,3,5,6]. The aim of treatment with the Ilizarov method, for orthopedists and patients alike, is to achieve best possible outcomes. Good Ilizarov treatment outcomes are associated with low complication rates, rapid formation of strong bone regenerate, and relatively early removal of the Ilizarov external fixator [1,2,3,9,10,11].

Some authors recommend the use of pharmaceutical agents that facilitate and accelerate bone union [12,13,14,15,16,17]. Such agents included calcium, vitamin D, bisphosphonates, some monoclonal antibodies, teriparatide and ossein–hydroxyapatite complex [12,13,14,15,16,17]. Some non-randomized studies involving small groups of patients have shown a beneficial effect of ossein–hydroxyapatite complex on fracture treatment outcomes and achieving bone union [15,16,17,18,19,20,21,22,23].

Most studies on Osteogenon (ossein–hydroxyapatite complex, Pierre Fabre, France) have been conducted in small, open-label, non-randomized designs, or in eastern Europe. Large, randomized controlled trials of this drug in orthopedic surgery are lacking, and the results regarding its analgesic effects are conflicting. A study assessing the potential positive impact of Osteogenon on patients after Ilizarov osteotomies is important for several reasons. Potentially, the use of this drug could shorten the duration of long-term Ilizarov treatment, reduce the high costs of Ilizarov treatment, reduce pain, and improve patients’ quality of life. Ilizarov osteotomies are quite common in Europe and worldwide, and Ilizarov treatment is associated with high costs ($30,000–$50,000 per procedure), partly due to the long-term nature of the procedure [4,6]. Evaluation of a drug that could potentially shorten treatment time, reduce the incidence of complications, and reduce the costs of treatment with the Ilizarov method, with a Taylor spatial frame or magnetic lengthening nails, is therefore important.

In our previous studies, we assessed the effect of Osteogenon on lower leg fracture treatment with the Ilizarov method and tibial nonunion treatment with the Ilizarov method [14,17]. We observed a beneficial effect of adjunctive use of Osteogenon in the treatment of lower leg fractures and nonunion with the Ilizarov method [14,17]. The nature of the Ilizarov method (which allows for fixator loosening prior to its planned removal, easy clinical assessment of bone union, and early fixator removal following bone union or formation of a sufficiently strong regenerate) facilitates precise evaluation for bone union and time to bone union [1,2,14,17]. Given the mechanism of action and properties of Osteogenon, and the similarity of callus formation following fracture and that of new bone (regenerate) formation following Ilizarov osteotomy, a beneficial effect of Osteogenon on Ilizarov osteotomy, arthrodesis or bone transport with the Ilizarov method outcomes seems plausible. Given the lack of extensive research on the ossein–hydroxyapatite complex, Osteogenon should be regarded as a dietary supplement containing this complex rather than as a drug with proven pharmacological efficacy.

There have been no studies to assess the effect of Osteogenon on the treatment outcomes of patients undergoing osteotomy with Ilizarov fixation. We hypothesized that Osteogenon would improve treatment outcomes in these patients. The purpose of our study was to assess radiological and clinical effects of Osteogenon in patients undergoing osteotomy combined with Ilizarov fixation.

## 2. Materials and Methods

### 2.1. Study Design and Patient Selection

In this retrospective study we assessed patients who had undergone lower leg lengthening osteotomies with the Ilizarov method in the years 2021–2022 and received adjunctive Osteogenon (ossein–hydroxyapatite complex). Study inclusion criteria were lower-leg lengthening osteotomy with the Ilizarov method, age > 18 years, complete medical and radiological records from the treatment period, a follow-up of over 6 months after treatment completion, the use of Osteogenon throughout the period of Ilizarov fixation, no use of other drugs affecting bone turnover, informed consent, no other musculoskeletal conditions, and no other conditions affecting bone tissue (including osteoporosis, osteomalacia, Paget disease, rickets, metabolic conditions). Study exclusion criteria included no history of lengthening lower-leg osteotomy with the Ilizarov method, incomplete medical and radiological records, age under 18 years, inconsistent or absent use of Osteogenon throughout the period of fixation, a follow-up period of less than 6 months after treatment completion, taking other drugs affecting bone turnover, a lack of informed consent, concomitant musculoskeletal conditions, or concomitant conditions of bone tissue. Patients who had undergone bone transport procedures or deformity corrections with lengthening were excluded from the study. The study had been approved by the local ethics committee and was conducted in accordance with the Declaration of Helsinki. All study participants were informed of the voluntary nature of their participation and the option of withdrawing their consent at any stage.

At our center, from 2021, all patients undergoing Ilizarov lower leg osteotomies routinely received Osteogenon. The operating physician prescribed Osteogenon to all patients following Ilizarov osteotomies at the time of hospital discharge. Patients also received prescriptions for Osteogenon during periodic follow-up visits at the orthopedic outpatient clinic. The decision to initiate Osteogenon treatment was not influenced by economic or clinical factors or by patient preferences. Patients from the experimental group received the drug Osteogenon (82 mg and 178 mg) twice a day throughout the period of treatment (from Ilizarov external fixator placement to its removal). The patients from the experimental group received no other drugs affecting bone union. Osteogenon has been approved for use in Poland in the treatment of fractures and as an adjunctive treatment of osteoporosis. Osteogenon contains 82 mg of phosphorus and 178 mg of calcium, insulin-like growth factors 1 and 2, beta transforming growth factor, and type I collagen [13,14,15,19].

Application of inclusion and exclusion criteria revealed 35 patients (15 males, 20 females; mean age 36 years [19–56]) to be eligible for further analysis. The control group comprised 60 patients (25 males, 35 females) after lower-leg lengthening osteotomy procedures performed with the Ilizarov method in the years 2018–2021 (we started using Osteogenon in 2021; prior to 2021, we did not use Osteogenon as an adjunct to the Ilizarov method) who did not receive Osteogenon, or any other drug altering bone metabolism, during treatment. All patients from both groups had been operated on by the same orthopedic surgeon using the same surgical technique. The two groups did not differ in terms of body mass index, age, sex distribution, the rate of smokers, or the rate of diabetic patients; see Table 1 and Table 2.

### 2.2. Treatment Protocol

The treatment protocol, follow-up visit and rehabilitation schedule was identical for each patient from either group. Despite the time differences (during the treatment period), there were no differences between the Osteogenon and control groups across the treatment regimen. There were no differences or changes between the groups in: team experience, antibiotic prophylaxis protocols, postoperative pain management, frequency of visits, and Ilizarov fixator removal criteria, among others. On postoperative day one, patients from both groups were made to get up and were educated on how to walk and bear weight on the treated limb without exceeding tolerable pain. The first outpatient follow-up visit involving a clinical and radiological assessment took place 14–16 days after the surgery. Subsequent outpatient visits were scheduled in 2-week intervals throughout the period of limb lengthening and deformity correction. During the regenerate consolidation phase, outpatient follow-up visits took place every 6 weeks. The patients were instructed to bear weight on the treated limb throughout the treatment period. The weight was gradually increased, within pain tolerance, until full weight bearing was achieved. Once radiological evidence of bone regenerate (i.e., at least 3 out of 4 cortices) was observed, the fixator was loosened. Subsequently, the patient walked for 7 days bearing full weight on the treated limb with the loosened fixator. After 7 days, a follow-up X-ray was taken, and–if no secondary deformities of the regenerate were observed–a clinical assessment of bone union was performed. Clinical evidence of bone union was positive if there was no pain or pathological mobility despite attempts at forcibly moving the limb and the loosened components of the Ilizarov fixator. Once clinical and radiological evidence of bone union was observed, the Ilizarov fixator was removed.

### 2.3. Outcome Measures

We assessed the following clinical and radiological parameters: the duration of fixation, total initial limb shortening, total limb lengthening, achieving bone union following osteotomy, lengthening index, alignment index, complications, analgesic use, and satisfaction with treatment. All clinical, radiological, and functional parameters were assessed retrospectively in all patients in both groups. Outcomes were assessed based on available medical and radiological documentation from the treatment and patient examinations with completed questionnaires at a follow-up visit at least 12 months after treatment completion. The radiological assessment was performed on the basis of telemetric X-ray images (measuring the lower limbs) and lateral X-ray images. The person performing the radiological assessment was blinded to the group.

The duration of fixation was expressed in days and defined as the time from the mounting to removal of the Ilizarov fixator. Achieving bone union was assessed based on clinical and radiological criteria [1,2,3,14,17]. The clinical criteria included lack of pain or pathological mobility at the osteotomy site despite attempts at forcible movement and bearing weight while walking with a loosened fixator [1,14,17]. The radiological criteria were a bridging callus across the whole bone regenerate and the presence of at least 3 of 4 cortices (anterior, posterior, lateral, medial cortical layers) [1,2,3,14,17].

The elongation index was the quotient of the number of days the Ilizarov fixator stayed mounted and the extent of limb lengthening expressed in centimeters [1,2,3]. The alignment index was defined as the quotient of limb lengthening (in cm) and the initial shortening (in cm) multiplied by 100%.

Complications were assessed based on a review of medical and radiological records and on a clinical examination. The following complications were assessed: pain, swelling, movement limitation, infections, soft tissue necrosis, vessel and nerve damage, loosening of the fixator, the need for reoperation, joint instability, nonunion, and premature union.

Satisfaction from treatment was assessed based on the subjective ratings of *very satisfied*, *satisfied*, or *unsatisfied*. We assessed the use of analgesic drugs, due to pain (ibuprofen, NSAIDs, opioids) in long-term follow-up. Complications, satisfaction from treatment and use of analgesic drugs were assessed based on patient interviews, medical record analysis, clinical examination findings, and an analysis of questionnaires completed during the long-term follow-up visit. In our study, the primary outcome measure was the elongation index (days/cm).

### 2.4. Statistical Analysis

Statistical analysis was performed using Statistica software (version 14.1). The distribution of continuous variables was assessed using the Shapiro–Wilk test. Due to non-normal distribution of the analyzed variables, data were expressed as medians and interquartile ranges (Q1–Q3), and comparisons between groups were conducted using the Mann–Whitney U test. Effect size for non-parametric comparisons was calculated as r (r = |Z|/√N), with approximate conversion to Cohen’s d reported for descriptive purposes. Categorical variables were presented as percentages and compared using the chi-square test. Effect size for categorical data was expressed as the φ coefficient (Cramér’s V for 2 × 2 tables). A *p*-value < 0.05 was considered statistically significant.

## 3. Results

The average follow-up period in the Osteogenon group was 32 months (6–49 months) and 70 months (51–88 months) in the control group. From 2018 to 2021, 72 Ilizarov lower leg osteotomies were performed. Twelve patients were excluded from the control group (six due to lack of complete medical and radiological documentation, four due to unwillingness to participate in the study, and two due to the use of other drugs affecting bone turnover). From 2021 to 2022, 44 Ilizarov lower leg osteotomies were performed in patients taking Osteogenon. Nine patients from the Osteogenon group were excluded from the study (five due to lack of complete medical and radiological documentation, two due to unwillingness to participate in the study, and two due to the use of other drugs affecting bone turnover). Detailed results of our studies for both groups are presented in Table 3 and Table 4.

**Figure 1:**
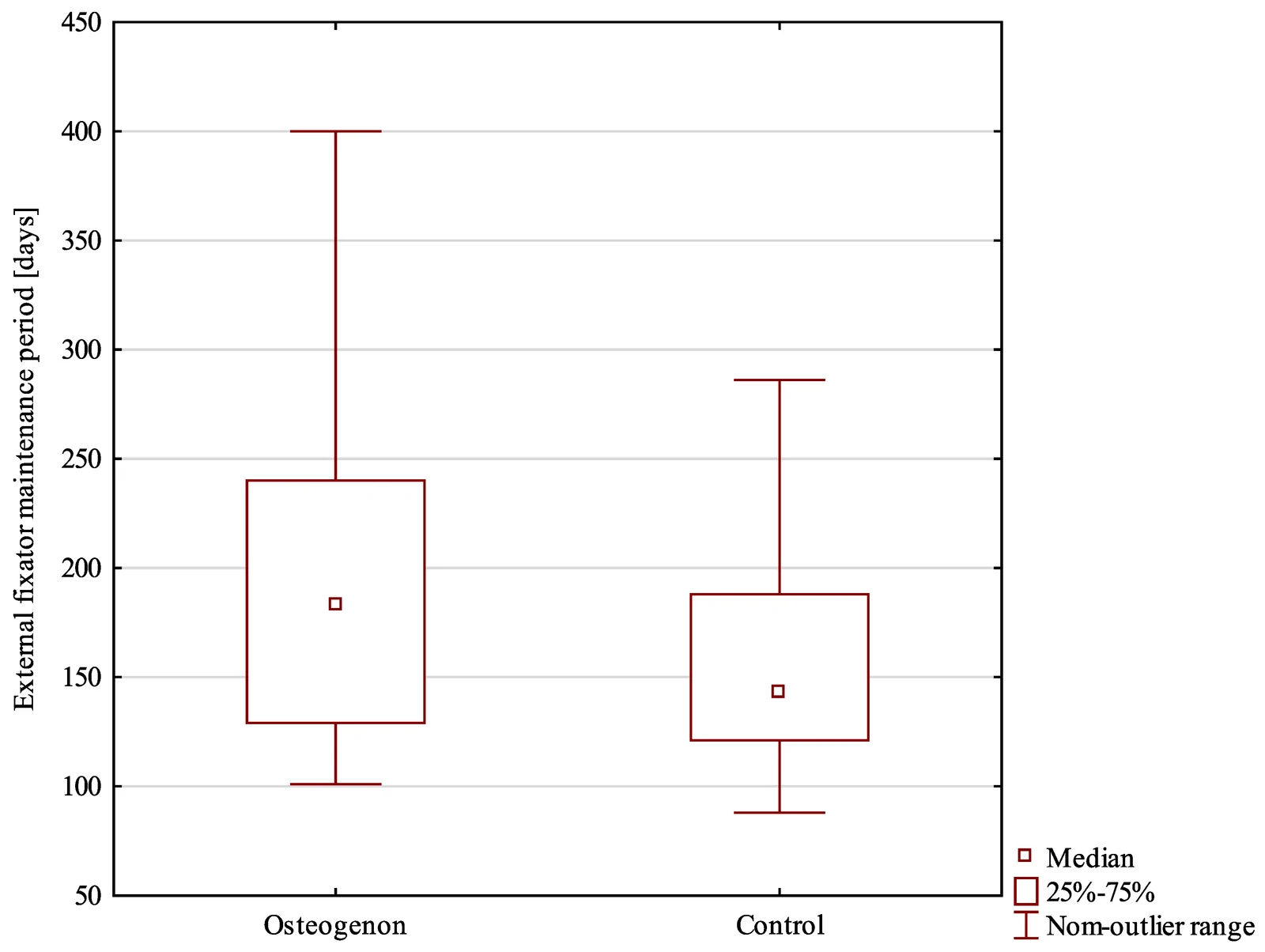
External fixator maintenance period in the Osteogenon group and in the control group.

The median duration of fixation was worse (longer) in the Osteogenon group (183 days) compared to the control group (143 days). There were statistical differences between study groups in fixation duration, with *p* = 0.014; see Figure 1 and Table 3.

**Figure 2:**
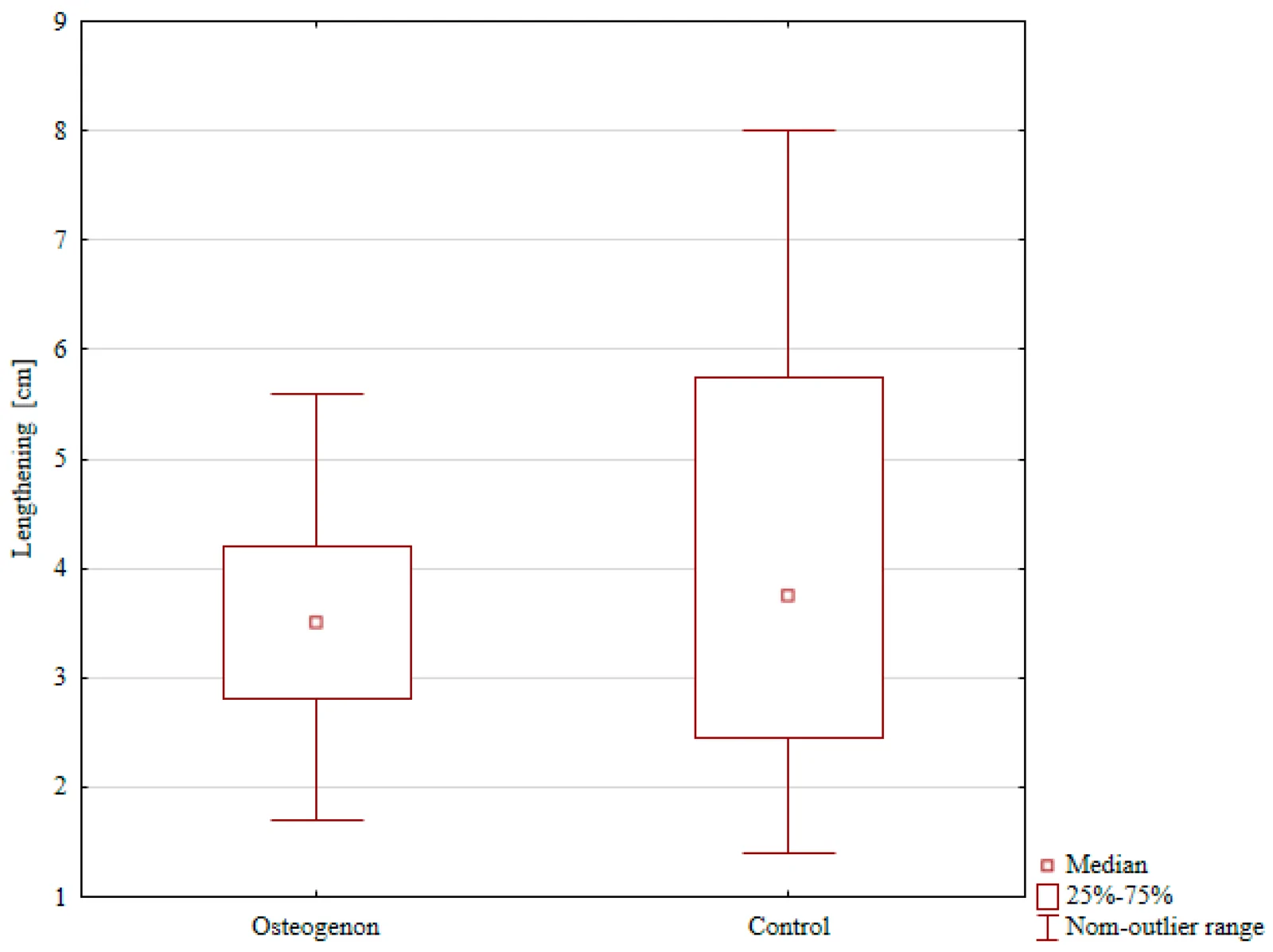
Limb lengthening in the Osteogenon group and in the control group.

The median initial limb shortening values in the Osteogenon and control groups were 3.5 cm (1.7–10.5 cm) and 4 cm (1.5–11.5 cm), respectively; the difference was not statistically significant; see Table 3. The median limb lengthening values in the Osteogenon and control groups were 3.5 cm (1.7–10.5 cm) and 3.75 cm (1.5–8 cm), respectively; this difference was not statistically significant; see Figure 2 and Table 3.

**Figure 3:**
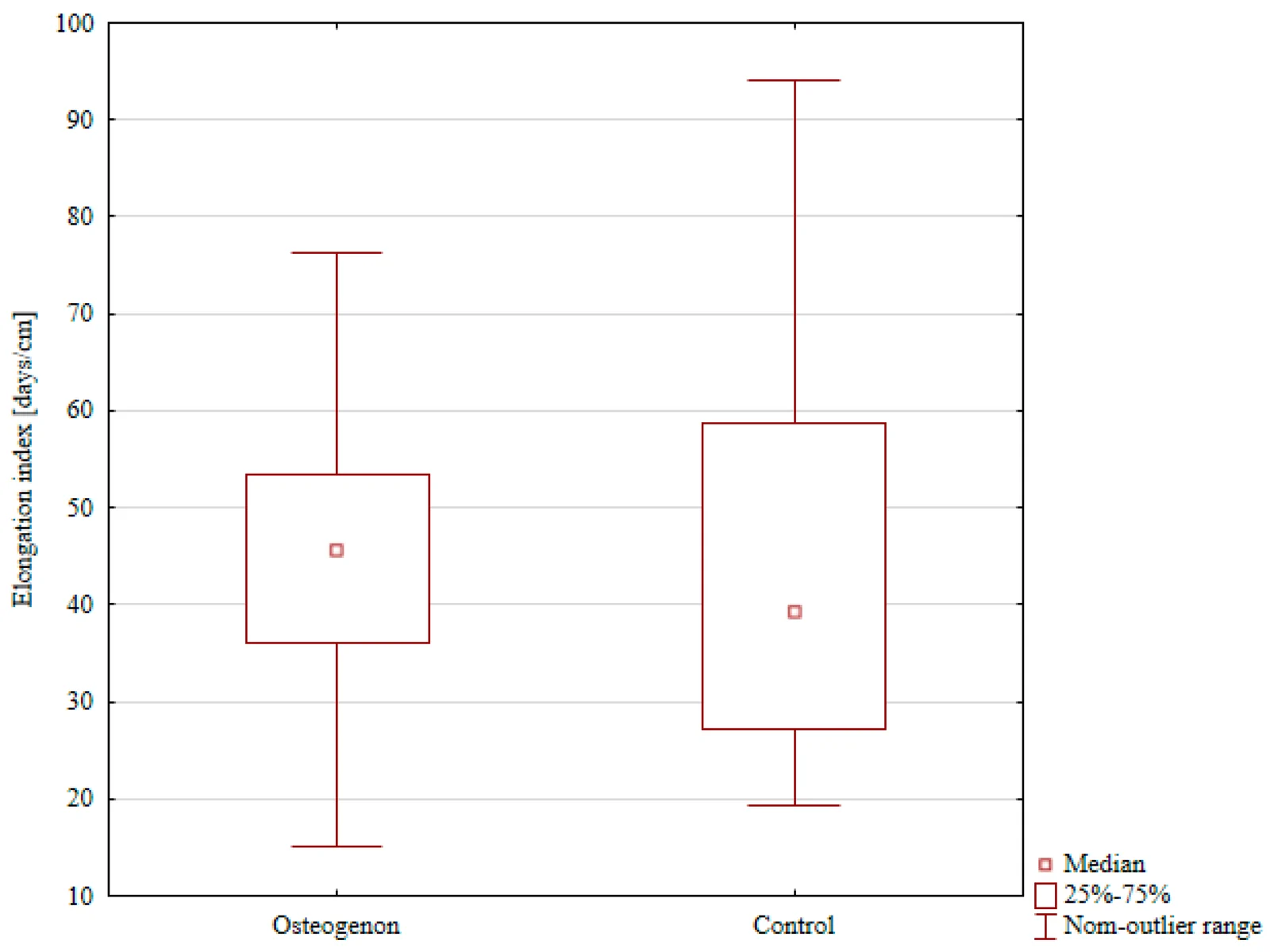
Elongation index in the Osteogenon group and in the control group.

The median elongation index in the Osteogenon and control groups were 48 days/cm and 39.16 days/cm, respectively; the difference was not statistically significant; see Figure 3 and Table 3.

**Figure 4:**
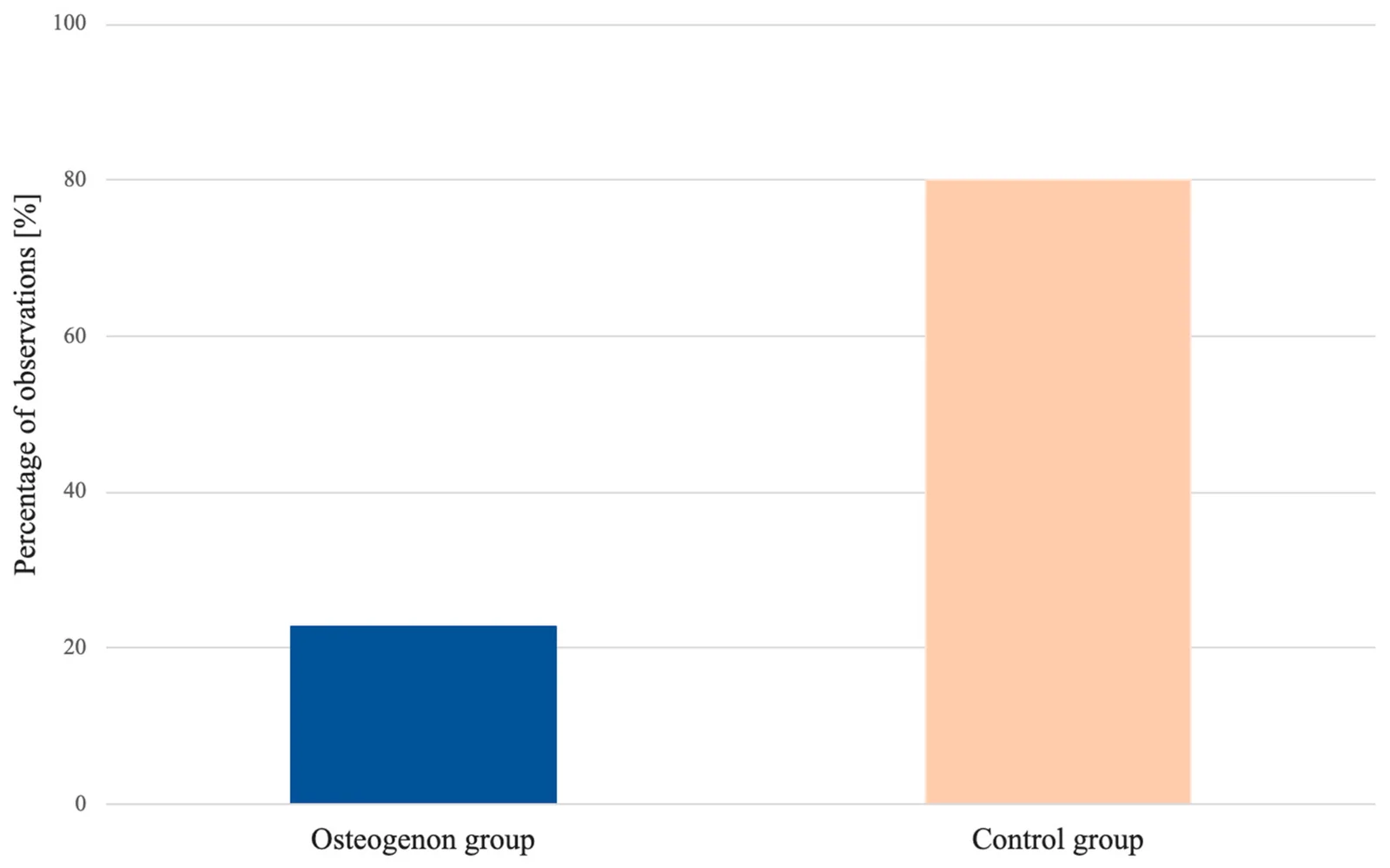
Percentage of patients on post-treatment follow-up taking analgesics from the Osteogenon group and the control group.

The median alignment index was 100% in both groups; see Table 3. Bone union was achieved in 100% of patients in both groups; see Table 4. When it comes to the use of analgesics on post-treatment follow-up, eight patients (22.9%) from the Osteogenon group and 48 patients (80%) from the control group used them; the difference was statistically significant; *p* < 0.001; see Figure 4 and Table 4.

A survey of treatment satisfaction in the Osteogenon group revealed 23 patients (65.71%) and 12 patients (34.29%) to be *very satisfied* and *satisfied* with treatment, respectively; there were no patients unsatisfied with treatment. In the control group, there were 33 patients (55%) and 25 patients (41.66%) *very satisfied* and *satisfied* with treatment, respectively, with two patients (3.33%) *unsatisfied*.

**Figure 5:**
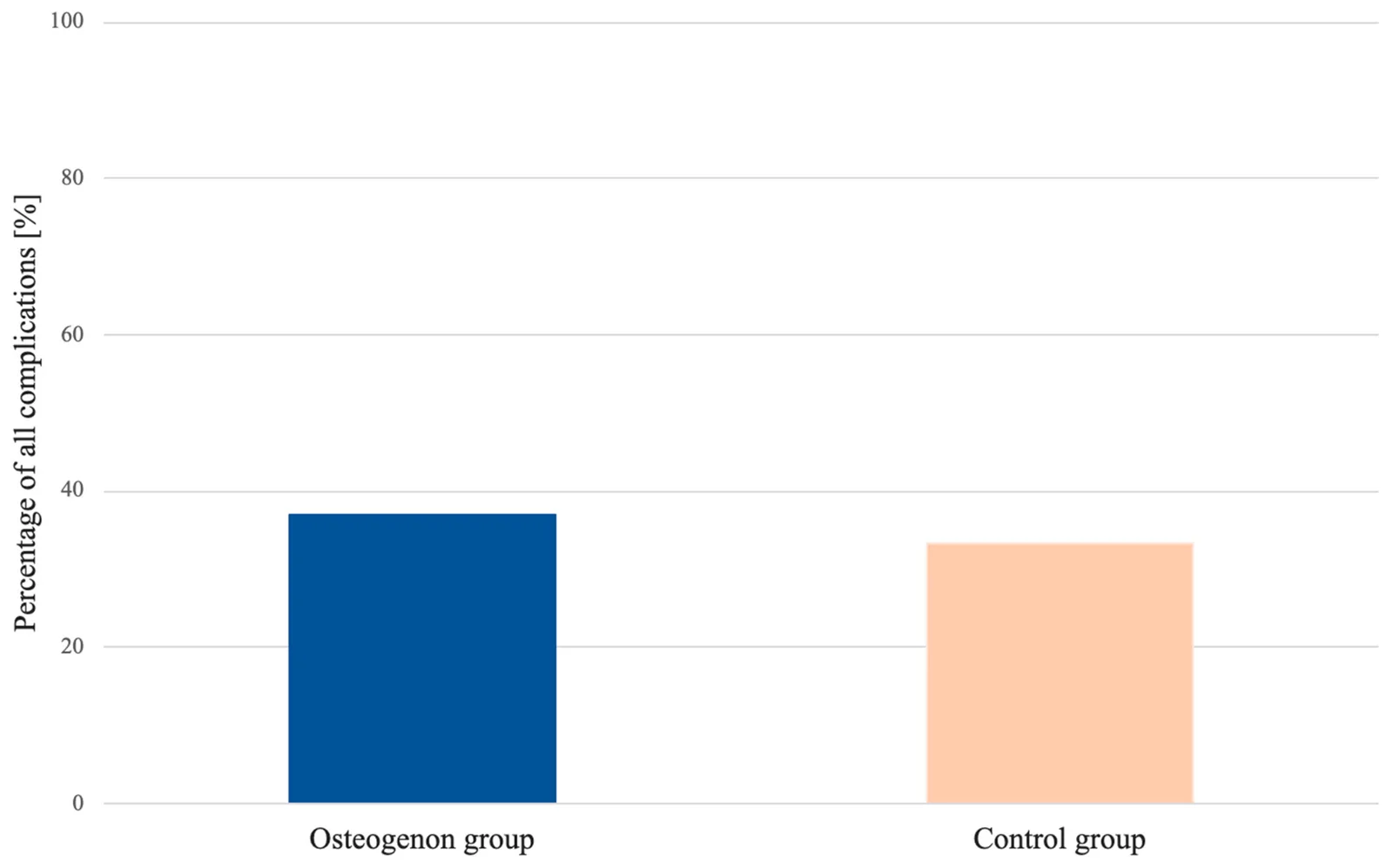
Percentage of patients with complications from the Osteogenon group and the control group.

Complications were reported by 13 patients (37.1%) from the Osteogenon group and 20 patients (33.3%) from the control group; the difference was not significant; see Figure 5 and Table 4.

In the Osteogenon group, nine patients (25.7%) exhibited limited joint motion, which was resolved after a longer and more intense rehabilitation, one patient (2.86%) experienced a broken Kirschner wire (requiring reconstructing the fixator without reoperation), and three patients (8.57%) developed superficial infections at the fixator mounting sites (which required dressing changes and oral antibiotic therapy). In the control group, 10 patients (16.7%) showed limited range of motion, which resolved after a longer and more intense rehabilitation, one patient (1.66%) experienced a broken Kirschner wire (which required fixator reconstruction without reoperation), three patients (5%) developed delayed bone union (requiring reoperation—involving Beck’s drilling method and administration of platelet-rich plasma), and six patients (10%) developed superficial infections around the fixator mounting sites (requiring dressing changes and oral antibiotic therapy).

## 4. Discussion

In this study, we assessed the use of Osteogenon as an adjunctive treatment in patients who underwent osteotomies with Ilizarov fixation. In our study, the primary outcome measure was the elongation index, which did not differ between the groups evaluated. Only two of the assessed parameters (subjective satisfaction with treatment and the number of patients using analgesics on follow-up) were better in the Osteogenon group than in the control group (not receiving Osteogenon). These results only partially support our research hypothesis.

There are few publications confirming the efficacy of the ossein–hydroxyapatite complex. Osteogenon should be regarded as a dietary supplement containing the ossein–hydroxyapatite complex rather than as a drug with proven pharmacological efficacy. The organic component of Osteogenon, ossein, activates osteoblasts and enhances their proliferation, accelerates callus formation, increases bone anabolism, and increases bone mass [12,16,17,18]. The inorganic component of the complex, hydroxyapatite, inhibits osteoclast proliferation and activity, which limits bone resorption, and constitutes a scaffolding for newly forming bone tissue [13,14,19,20,21]. The commercially available ossein–hydroxyapatite complex formulation (Osteogenon) additionally contains type I collagen, beta-transforming growth factor, and insulin-like growth factors 1 and 2 [14,15,16,19,20,21,22,23]. Studies showed Osteogenon to increase mineral bone density, to accelerate bone union following fractures, and to accelerate cortical bone formation [12,13,14,15,16,20].

Studies analyzing the bioavailability and pharmacokinetic properties of orally administered insulin-like growth factors 1 and 2 have shown that high serum concentrations of these compounds are maintained for up to 48 h post-administration (with peak concentrations reached after 4–8 h); this phenomenon was also observed in patients with renal insufficiency [24,25,26]. It has been demonstrated that following the oral administration of transforming growth factor-beta, its serum concentration remains elevated for up to 24 h, reaching a maximum value approximately 3 h after administration [27,28]. Complex proteins are generally degraded within the gastrointestinal tract and do not reach systemic circulation in biologically relevant concentrations. Therefore, the proposed mechanism of action should be critically analyzed and approached with caution. To date, there are no dedicated studies evaluating the pharmacokinetics or bioavailability of Osteogenon.

A strong and sturdy bone regenerate, its rapid turnover, and the shortest possible duration of Ilizarov fixation are important both for patients and for surgeons. Some of the factors affecting good treatment outcomes in patients undergoing osteotomies with Ilizarov fixation are short durations of fixation, low complication rates, and a low lengthening index [1,2,3,7,10,11,29,30,31]. Slow bone regenerate formation or a poor-quality regenerate are among the most common problems with Ilizarov-method treatment [5,8]. In this study, we hypothesized that ossein–hydroxyapatite complex properties, including osteoblast activation, osteoclast suppression, and a beneficial effect on osteogenesis [12,16,18,19,20,21,22,23], would have a positive impact on the process of bone regenerate formation following osteotomy combined with Ilizarov external fixation. The ossein–hydroxyapatite complex may have potentially positive effects in patients after arthrodesis and bone transport with the Ilizarov method or in patients after osteotomies using magnetic lengthening nails or Taylor spatial frames. Other positive aspects of ossein–hydroxyapatite complex, which might improve Ilizarov treatment outcomes, have also been demonstrated. These include accelerated callus formation, accelerated cortical bone formation, increased calcium levels, and an increased bone mineral density (BMD) [13,14,15,19,20,21,22,23]. The lack of analgesic use and the patient’s subjective satisfaction with treatment might also be considered in treatment outcome assessment [13,14,16,17,18].

Bisphosphonates, some monoclonal antibodies, teriparatide, and antisclerostin therapies also have a positive effect on bone healing and bone remodeling after fractures [12,13,14,15,16,17]. Several of these interventions have been evaluated in randomized controlled trials and provide a stronger level of evidence regarding fracture healing and callus formation [32,33,34]. Studies assessing the effect of ossein–hydroxyapatite complex on fractures and osteoporosis have been conducted in small, open-label, non-randomized designs, or in eastern Europe.

In a group of 50 patients evaluated by Makhdoom et al. the mean elongation index was 1.6 months/cm [1]. Depaoli et al. reported a mean elongation index of 1.83 months/cm in children who underwent lower-leg osteotomies [2]. Those authors concluded that complications may increase the lengthening index [2].

For a group of 27 patients who underwent lower-leg lengthening with the Ilizarov method in a study by Kristiansen, the mean elongation index was 1.8 months/cm [3]. The mean elongation index in patients assessed by Matsubara et al. was 43 days/cm [5]. Patients with congenital limb shortening and deformity treated with Ilizarov-method osteotomies showed a mean elongation index of 50.7 days/cm [6]. The mean elongation index in a group of 24 adults assessed by Armagan et al. was 63.7 days/cm [7]. Popkov et al. reported a mean elongation index of 32.5 days/cm [8], whereas another study showed the mean value of this parameter to be 34.7 days/cm [9]. The patients who underwent osteotomies combined with Ilizarov fixation as part of the study by Bukva et al. showed a mean elongation index of 43.7 days/cm [11]. Kristiansen and Steen evaluated a monofocal tibial osteotomy on 31 segments and found an average elongation index of 51 days/cm [29]. In the group of patients after lower leg osteotomies, assessed by Lan et al., the mean elongation index was 76.19 days/cm [30].

The mean elongation index in patients assessed in our study did not differ between the study groups and was comparable with those reported in the literature [1,2,3,5,6,7,8,9,10,11,29,30]. These results show that the use of Osteogenon had no effect on the elongation index (which was the primary outcome measures) in our patients.

Gulnazurova and Kuznetsova assessed the effect of ossein–hydroxyapatite complex on the treatment of fracture nonunion [12]. Those authors reported achieving bone union in all evaluated patients and shortening of the consolidation phase by 2–3 months, in comparison with that in the control group, who did not receive ossein–hydroxyapatite complex [12]. All patients from our Osteogenon group achieved bone union, as did all patients from our control group. Makhdoom reported bone union in all patients who underwent osteotomies [1]. Our study outcomes indicate a lack of effect of ossein–hydroxyapatite complex on the proportion of achieved bone unions following lower-leg osteotomies combined with Ilizarov fixation.

The duration of fixation in the Osteogenon group was significantly longer than that in the control group; however, this did not affect inter-group differences in terms of the lengthening index or alignment index. We are unable to determine why the duration of fixation was longer in the group receiving Osteogenon, which potentially has a positive effect on bone formation, than in the control group. This could potentially be due to different etiologies of the shortening; however, the literature reports indicate that etiology has no impact on the lengthening index, complication rates, or other outcomes of Ilizarov-method treatment [1,2]. The duration of fixation in both assessed groups was comparable to, or somewhat shorter than, those reported by other authors [2,7,8,10,11].

In our study, the alignment index was 100% in either group, which shows a lack of effect of the use of Osteogenon on this parameter. All patients in the Osteogenon group were satisfied or very satisfied with treatment; whereas patients from the control group reported a worse subjective rating of treatment satisfaction. This may have resulted from the fact that a much lower proportion of patients from the Osteogenon group felt pain after surgery and continued to take analgesic drugs after treatment completion.

Pain may limit mobility and adversely affect the mental health of patients, causing depression and a need to isolate from others [18]. On follow-up one year after treatment initiation, Rodionova et al. reported pain relief in 87% of patients receiving Osteogenon and in 50% of patients not receiving the drug [13]. Varga et al. demonstrated a significant effect of ossein–hydroxyapatite complex on pain intensity visual-analog-scale (VAS) scores in patients with radius fractures [16]. Another study showed a significant effect of ossein–hydroxyapatite complex on reducing back and knee pain in women with osteoporosis [18]. On the other hand, Osteogenon had no effect on the number of patients with pain relief following lower-leg fracture treatment with the Ilizarov method or following tibial nonunion treatment with the Ilizarov method [14,17].

In our study, only 22.9% of patients from the Osteogenon group and as many as 80% of patients from the control group were taking analgesics on post-treatment follow-up. This indicates a good effect of Osteogenon after lower-leg osteotomies with the Ilizarov method. This lower proportion of patients in the Osteogenon group taking analgesic agents may be due to intrinsic analgesic properties of Osteogenon [12,13,16,17,18,20]. The mechanism of analgesic action of Osteogenon is not fully understood [12,13,16,17,18] and may be a result of osteoclast suppression, since osteoclasts are involved in processing pain [18]. Another theory posits that the growth factors present in Osteogenon play a role in the perception of pain [18]. The limited use of painkillers in the group receiving Osteogenon is noteworthy. However, given the lack of randomization and blinding, this result is highly susceptible to the placebo effect and expectation bias. In the absence of blinding, reduced analgesic use may stem from treatment-related expectations rather than an actual biological effect. In summary, the available data appear to point more towards a possible placebo effect than to a measurable improvement in the bone healing process.

Despite the ever-improving surgical techniques, the rates of complications in patients treated with the Ilizarov method remain high [1,2,3,7,9,10]. According to some reports, a longer duration of fixation may increase the risk of complications [7,8]; therefore, we should strive to remove the Ilizarov fixator as soon as possible following bone regenerate formation. Theoretically, such pharmaceutical agents as Osteogenon might accelerate the time to achieve bone union based on regenerate formation and shorten the time the Ilizarov fixator remains mounted. However, the results of our study do not support such effects of Osteogenon.

Makhdoom reported 12% of patients who underwent osteotomies combined with Ilizarov fixation having developed complications (incomplete osteotomy or fractured bone) [1]. Out of 178 children who underwent femoral and lower-leg osteotomies with the Ilizarov method, 79% developed complications [2]. In a group of 27 patients who underwent the same treatment, Kristianse reported complications in 100% of cases [3]. In the group treated by Armagan et al. 100% of patients developed complications [7]. Emara also reported complications in all treated patients [9]. Krappinger observed complications in 50% of patients [10]. Aaron and Eilert found complications in 72% of patients following Ilizarov osteotomies [31].

In our study, a comparable proportion of patients from the Osteogenon and control groups developed complications. The complication rates in our study were somewhat lower than those reported by other authors [1,2,3,7,9,10,31]. Our study results suggest a lack of effect of ossein–hydroxyapatite complex on the number of complications in patients undergoing lower-leg osteotomy combined with Ilizarov fixation.

Osteogenon is a well-tolerated drug, with reported side effects affecting only up to 3.2% of patients [12,13,14,15,16,17,19]. In our study there were no side effects of using Osteogenon. Contraindications for the use of this drug include age under 18 years, calcium-based kidney stones, chronic kidney disease, hypercalciuria, dialysis therapy, and hypercalcemia. We did not stratify our patients by etiology; however, the literature reports indicate that etiology has no impact on the lengthening index, complication rates, or other outcomes of Ilizarov-method treatment [1,2].

The follow-up period for the Osteogenon and control groups differed. Despite the differences in follow-up period between groups, there were no other significant differences in medical and epidemiological parameters between groups that could have confounded the results. All patients from both groups had been operated on by the same orthopedic surgeon using the same surgical technique. The treatment protocol, follow-up visit and rehabilitation schedule was identical for each patient from either group. There were no differences or changes between the groups in: team experience, antibiotic prophylaxis protocols, postoperative pain management, frequency of visits, and Ilizarov fixator removal criteria, among others. Generalizability is also limited by the involvement of a single surgeon, the exclusively Polish study population, and the inclusion of mixed, unstratified etiologies. Armagan et al. report that patient age may influence the number of complications and the duration of fixator maintenance following Ilizarov osteotomies [7]. The patients in our study did not differ in age across both groups, which is a strength of our study.

The limitations of our study include its retrospective nature, which results from our intention to include large study and control populations and our aim to present the study results relatively quickly. Attention should also be paid to the issue of generalization (only one surgeon, Polish population, mixed etiologies, unstratified). Another limitation of our study was the fact that no bone density scans were conducted; this was a result of this assessment not having been available, missing bone density scan results in a majority of our patients, and the lack of funds for these assessments. Potential limitations of the study include different observation periods (although the treatment protocol was the same in both groups), lack of blinding in subjective assessments, unvalidated outcome satisfaction, and lack of adjustment for multiple comparisons. Studies on the effects of Osteogenon on osteoblast activation and osteoclast inhibition were previously conducted by other researchers cited in our manuscript. In our manuscript, we focused on assessing clinical, radiological, and functional parameters in patients after Ilizarov osteotomies. Due to lack of funding and resources, we did not evaluate biochemical parameters (e.g., alkaline phosphatase, osteocalcin, etc.). We believe that the simultaneous assessment of clinical, radiological, functional, and biochemical markers in a single manuscript hindered the reading and interpretation of the data and resulted in data redundancy. Future analysis of biochemical markers of bone metabolism in patients treated with the Ilizarov method and receiving Osteogenon is recommended. One of the limitations of our study was the lack of randomization. This was due to the desire to present our findings quickly and the retrospective nature of the study. Some existing publications regarding the ossein–hydroxyapatite complex also lack randomization. In the future, we plan to conduct similar prospective, randomized studies.

One strength of our study is a relatively large sample size, comparable body-mass-index, sex, and age distribution between study groups, and comparable comorbidity rates between study groups. Other strengths of our study include an identical surgery protocol and treatment and rehabilitation regimen for each patient, and each operation having been performed by the same orthopedic surgeon. Our patients were heterogeneous in terms of surgical procedures. In the future we are planning similar, but prospective, studies to assess more clinical and functional parameters, and to include bone density scanning. We ourselves are surprised that Osteogenon increases the external fixator maintenance period.

The use of Osteogenon does not significantly improve treatment outcomes in patients with lower-leg osteotomy combined with Ilizarov fixation. Taking Osteogenon improves patients’ satisfaction with treatment and reduces analgesic use following osteotomies combined with Ilizarov fixation. However, Osteogenon after Ilizarov osteotomies should be used with caution, as it increases the external fixator maintenance period.

## 5. Conclusions

Taking Osteogenon improves patient’s satisfaction with treatment and limits analgesic use following osteotomies combined with Ilizarov fixation.

Osteogenon was associated with a significantly longer external fixation period and failed to demonstrate measurable improvements in radiological outcomes.

Any considerations regarding the potential benefits of using Osteogenon should be treated with caution.

The use of Osteogenon in Ilizarov osteotomies does not adequately meet research objectives—it does not significantly impact public health.

## Figures and Tables

**Table 1:** Continuous demographic variables of the Osteogenon and control groups.

Analyzed Variable	Group	Q1	Median	Q3	Z	*p*	Effect Size
Age [years]	Osteogenon	28.0	33.0	44.0	Z = 0.096	0.923	r = 0.010
	Control	26.0	33.5	43.0			
BMI [kg/m^2^]	Osteogenon	22.87	29.11	31.90	Z = 0.305	0.761	r = 0.031
	Control	22.87	28.19	31.54			

Z—standardized value of the Mann–Whitney U test; *p*—*p*-value. Q1, Q3—1st and 3rd quartile. r—effect size for the Mann–Whitney test (r = |Z|/√N).

**Table 2:** Categorical demographic variables of the Osteogenon and control groups.

Analyzed Variable	Group	% of Observations	χ^2^	*p*	Effect Size
Male	Osteogenon	42.9%	Χ^2^ = 0.013	0.910	φ = 0.012
	Control	41.7%			
Smokers	Osteogenon	11.4%	χ^2^ = 0.048	0.827	φ = 0.022
	Control	10.0%			
Diabetes	Osteogenon	11.4%	χ^2^ = 0.048	0.827	φ = 0.022
	Control	10.0%			

χ^2^—chi-square test statistic; *p*—*p*-value. φ—effect size for the chi-square test (Cramér’s V for 2 × 2 tables).

**Table 3:** Continuous clinical and radiological parameters of the Osteogenon and control groups.

Analyzed Variable	Group	Q1	Median	Q3	Z	*p*	Effect Size
External fixator maintenance period [days]	Osteogenon	129.0	183.0	240.0	Z = 2.470	0.014	r = 0.273
	Control	121.0	143.0	188.0			
Initial shortening [cm]	Osteogenon	3.0	3.5	4.8	Z = −0.662	0.508	r = 0.071
	Control	2.65	4.0	6.0			
Lengthening [cm]	Osteogenon	3.0	3.5	4.2	Z = −0.165	0.869	r = 0.018
	Control	2.45	3.75	5.75			
Elongation index [days/cm]	Osteogenon	40.0	48.0	56.2	Z = 1.269	0.204	r = 0.165
	Control	27.2	39.16	58.75			
Alignment index [%]	Osteogenon	100.0	100.0	100.0	Z = 1.076	0.282	r = 0.110
	Control	100.0	100.0	100.0			

Z—standardized value of the Mann–Whitney U test; *p*—*p*-value; Q1, Q3—1st and 3rd quartile; r—effect size for the Mann–Whitney test (r = |Z|/√N).

**Table 4:** Categorical clinical outcomes of the Osteogenon and control groups.

Analyzed Variable	Group	% of Observations	χ^2^	*p*	Effect Size
Taking analgesics in the post-treatment follow-up	Osteogenon	22.9%	χ^2^ = 31.225	<0.001	φ = 0.573
	Control	80.0%			
Bone union	Osteogenon	100.0%	χ^2^ = 0.000	1.000	φ = 0.000
	Control	100.0%			
All complications	Osteogenon	37.1%	χ^2^ = 0.142	0.707	φ = 0.039
	Control	33.3%			
Delayed bone union	Osteogenon	0.0%	χ^2^ = 1.807	0.179	φ = 0.138
	Control	5.0%			
Limitation of joint mobility	Osteogenon	25.7%	χ^2^ = 1.131	0.288	φ = 0.109
	Control	16.7%			

χ^2^—chi-square test statistic; *p*—*p*-value; φ—effect size for the chi-square test (Cramér’s V for 2 × 2 tables).

## Data Availability

The data presented in this study are available on request from the corresponding author.

## References

[1] Makhdoom A., Kumar J., Siddiqui A.A. (2019). Ilizarov External Fixation: Percutaneous Gigli Saw Versus Multiple Drill-hole Osteotomy Techniques for Distraction Osteogenesis. Cureus.

[2] Depaoli A., Magnani M., Casamenti A., Cerasoli T., Ramella M., Menozzi G.C., Mordenti M., Rocca G., Trisolino G. (2023). Is the High Healing Index a Complication of Progressive Long Bone Lengthening? Observations from a Cohort of 178 Children Treated with Circular External Fixation for Lower Limb Length Discrepancy. Children.

[3] Kristiansen L.P., Steen H., Reikerås O. (2006). No difference in tibial lengthening index by use of Taylor spatial frame or Ilizarov external fixator. Acta Orthop..

[4] Dvorzhinskiy A., Zhang D.T., Fragomen A.T., Rozbruch R. (2021). Cost Comparison of Tibial Distraction Osteogenesis Using External Lengthening and Then Nailing vs Internal Magnetic Lengthening Nails. Strateg. Trauma Limb Reconstr..

[5] Matsubara H., Tsuchiya H., Sakurakichi K., Watanabe K., Tomita K. (2006). Deformity correction and lengthening of lower legs with an external fixator. Int. Orthop..

[6] Williams M.O. (1994). Long-term cost comparison of major limb salvage using the Ilizarov method versus amputation. Clin. Orthop. Relat. Res..

[7] Armagan R., Kucukkaya M., Ozdemir H.M. (2023). The Use of Ilizarov Method at the Lower Extremity Deformity Management. Sisli Etfal Hastan. Tip. Bul..

[8] Popkov A., Aranovich A., Antonov A., Journeau P., Lascombes P., Popkov D. (2020). Lower limb lengthening and deformity correction in polyostotic fibrous dysplasia using external fixation and flexible intramedullary nailing. J. Orthop..

[9] Emara K., Farouk A., Diab R. (2011). Ilizarov technique of lengthening and then nailing for height increase. J. Orthop. Surg..

[10] Krappinger D., Zegg M., Smekal V., Huber B. (2014). Correction of posttraumatic lower leg deformities using the Taylor Spatial Frame. Oper. Orthop. Traumatol..

[11] Bukva B., Brdar R., Nikolic D., Petronic I., Ducic S., Abramovic D. (2013). Combined external fixation and intramedullary alignment in correction of limb length discrepancies. Acta Orthop. Belg..

[12] Gulnazurova S.V., Kuznetsova O.A. (2006). Ossein-hydroxyapatite complex in the treatment of patients with pseudoarthrosis of the femur and shin bones, complicated by systemic osteoporosis. Bull. Traum.

[13] Rodionova S.S., Kolondaev A., Sokolov V.A., Markov S. (2001). Experience with the use of osteogenon in traumatology and orthopedics. NWN Priorov J. Traumatol. Orthop..

[14] Morasiewicz P., Zaborska M., Sobczak M., Tomczyk Ł., Leyko P., Bobiński A., Kochańska-Bieri J., Pili D., Kazubski K. (2024). The Use of Osteogenon as an Adjunctive Treatment in Lower Leg Fractures. Pharmaceuticals.

[15] Ciria-Recasens M., Blanch-Rubió J., Coll-Batet M., Lisbona-Pérez M.d.P., Díez-Perez A., Carbonell-Abelló J., Manasanch J., Pérez-Edo L. (2011). Comparison of the effects of ossein-hydroxyapatite complex and calcium carbonate on bone metabolism in women with senile osteoporosis: A randomized, open-label, parallel-group, controlled, prospective study. Clin. Drug Investig..

[16] Varga O., Vezikova N.N., Marusenko I.M., Kheĭfets L.M. (2007). Osteogenon in therapy of distal radial bone fractures in patients with secondary osteoporosis. Ter. Arkh..

[17] Morasiewicz P., Zaborska M., Sobczak M., Tomczyk Ł., Pili D., Kazubski K., Leyko P. (2025). The Use of Ossein-Hydroxyapatite Complex in Conjunction with the Ilizarov Method in the Treatment of Tibial Nonunion. J. Clin. Med..

[18] Castelo-Branco C., Dávila J., Alvarez L., Balasch J. (2015). Comparison of the effects of calcium carbonate and ossein-hydroxyapatite complex on back and knee pain and quality of life in osteopenic perimenopausal women. Maturitas.

[19] Castelo-Branco C., Cancelo Hidalgo M.J., Palacios S., Ciria-Recasens M., Fernández-Pareja A., Carbonell-Abella C., Manasanch J., Haya-Palazuelos J. (2020). Efficacy and safety of ossein-hydroxyapatite complex versus calcium carbonate to prevent bone loss. Climacteric.

[20] Yakovleva O., Hoina-Kardasevich O., Shcherbeniuk N. (2023). Efficacy of Ossein-Hydroxyapatite Complex as a Pharmacological Corrector of Bone Loss (Review). Georgian Med. News.

[21] Malachowski K., Materkowski M. (2020). Osteoporotic Fractures During and after Pandemic Time—Always Required Treatment. Approach of the Physician, Comments of the Professor. Ortop. Traumatol. Rehabil..

[22] Castelo-Branco C., Dávila Guardia J. (2015). Use of ossein-hydroxyapatite complex in the prevention of bone loss: A review. Climacteric.

[23] Fernández-Pareja A., Hernández-Blanco E., Pérez-Maceda J.M., Riera Rubio V.J., Palazuelos J.H., Dalmau J.M. (2007). Prevention of osteoporosis: Four-year follow-up of a cohort of postmenopausal women treated with an ossein-hydroxyapatite compound. Clin. Drug Investig..

[24] Park J., Yan G., Kwon K.-C., Liu M., Gonnella P., Yang S., Daniell H. (2019). Oral delivery of novel human IGF-1 bioencapsulated in lettuce cells promotes musculoskeletal cell proliferation, differentiation and diabetic fracture healing. Biomaterials.

[25] Rabkin R., Fervenza F.C., Maidment H., Ike J., Hintz R., Liu F., Bloedow D.C., Hoffman A.R., Gesundheit N. (1996). Pharmacokinetics of insulin-like growth factor-1 in advanced chronic renal failure. Kidney Int..

[26] Young S.C., Miles M.V., Clemmons D.R. (1992). Determination of the pharmacokinetic profiles of insulin-like growth factor binding proteins-1 and -2 in rats. Endocrinology.

[27] Hammer L., Furtado S., Mathiowitz E., Auci D.L. (2020). Oral encapsulated transforming growth factor β1 reduces endogenous levels: Effect on inflammatory bowel disease. World J. Gastrointest. Pharmacol. Ther..

[28] Zioncheck T.F., Chen S.A., Richardson L., Mora-Worms M., Lucas C., Lewis D., Green J.D., Mordenti J. (1994). Pharmacokinetics and tissue distribution of recombinant human transforming growth factor beta1 after topical and intravenous administration in male rats. Pharm. Res..

[29] Kristiansen L.P., Steen H. (2002). Reduced lengthening index by use of bifocal osteotomy in the tibia: Comparison of monofocal and bifocal procedures with the Ilizarov external fixator. Acta Orthop. Scand..

[30] Lan X., Zhang L., Tang P., Xia H., Li G., Peng A., Han Y., Yuan B., Xu W. (2013). S-osteotomy with lengthening and then nailing compared with traditional Ilizarov method. Int. Orthop..

[31] Aaron A.D., Eilert R.E. (1996). Results of the Wagner and Ilizarov methods of limb-lengthening. J. Bone Jt. Surg. Am..

[32] Händel M.N., Cardoso I., von Bülow C., Rohde J.F., Ussing A., Nielsen S.M., Christensen R., Body J.J., Brandi M.L., Diez-Perez A. (2023). Fracture risk reduction and safety by osteoporosis treatment compared with placebo or active comparator in postmenopausal women: Systematic review, network meta-analysis, and meta-regression analysis of randomised clinical trials. BMJ.

[33] Ayers C., Kansagara D., Lazur B., Fu R., Kwon A., Harrod C. (2023). Effectiveness and Safety of Treatments to Prevent Fractures in People with Low Bone Mass or Primary Osteoporosis: A Living Systematic Review and Network Meta-analysis for the American College of Physicians. Ann. Intern. Med..

[34] Li M., Ge Z., Zhang B., Sun L., Wang Z., Zou T., Chen Q. (2024). Efficacy and safety of teriparatide vs. bisphosphonates and denosumab vs. bisphosphonates in osteoporosis not previously treated with bisphosphonates: A systematic review and meta-analysis of randomized controlled trials. Arch. Osteoporos..

